# An Essay—Read before the Atlanta Medical Society

**Published:** 1859-03

**Authors:** Hayden Coe

**Affiliations:** Atlanta, Ga.


ATLANTA

### Medical and Surgical Journal.

VOL. IV.] MARCH, 1859. [NO. VII.

### ORIGINAL COMMUNICATIONS.

### ARTICLE I.

*An Essay—read before the Atlanta Medical Society by* HAYDEN COE, M. D. *Atlanta, Ga.*

*Gentlemen*—I wish to call your attention for a few moments to the importance of the study and investigation of the human system; not only by men of our profession, but by the whole human family, and I will offer you a few reasons why it is deserving of more attention than it has hitherto received.

It is true that there have been many individuals who have devoted their time, and their talents, to the study and investigation of the human system; some men, too, who have been distinguished for their talents.

But it must and will be admitted, that by far the larger portion of the human family are ignorant, yes, grossly ignorant of the nature and operations of their own systems; and it is greatly to be feared, that too many men of our own profession are too ignorant of the nature and operation of the different organs of the human system, which they profess to understand, and to have the skill to regulate.

Why it is, that a subject which seems to be so closely allied to man's best interest, one, when investigated, that is to be so much admired, one that must be and will be admitted by all, is of vast importance, should have received so little attention from the mass of mankind, is a mystery which I have never been able, as yet, to solve.

When we have taken a survey of the vast and almost innumerable variety of inventions which have been accomplished by the ingenuity of man, we have found many which we have beheld with admiration and wonder, and studied and investigated the nature and workings of every part with intense interest and delight. Yet I ask, where can you put your hand upon a piece of mechanism invented by the ingenuity of man, that is so much to be admired as your own body? how wonderfully made! Examine the beautiful mechanism of the eye—look at your own hand, how wonderfully formed; and its performance is still more wonderful; directed by and under the control of the will, its actions are almost innumerable. Examine the organs of articulation; the voice, with its countless number and variety of motions. I might go on to call your attention to the wonderful performance of the muscular system, by which all our actions are performed; and the glandular system which has the power of selecting from the blood those particles which are no longer necessary for the nourishment and support of the system, but would even act as a poison if retained, thereby removing them from the system.

I might speak of the wonderful power of the absorbent system; a system of which over one half the human family have never heard. If they have even heard of it, they have no idea what kind of a looking thing it is, and still less idea of its operations. I might speak of the nervous system—that system from which arises all our pleasure and all our pain, composed as it is of two parts, a centre from which proceed delicate and thread-like cords, to every part of the system, to carry impressions to and from the great centre which presides over all our actions.

Gentlemen, were you to survey God's universe, where, I ask, would you find an object so much to be admired, so wonderfully made, as your own body? made by God himself, more than five thousand years ago, from the inanimate dust of the earth, who has placed in that body a living soul, to preside over its actions, with laws to govern it, which laws cannot be violated with impunity. Yes, gentlemen, in the formation of your body is displayed the wisdom of a God. Yet how negligent has mankind been in the study and investigation of their own systems, as if there was something unpleasant, or even horrid, in one's knowing the operations of his own system. While men have fathomed the depth of the ocean, and have attempted to penetrate to the very centre of the earth, and have even tried to discover the composition of the moon, and to distinguish its inhabitants, and ascertain the laws by which they were governed, and have tried to discover new worlds, and the laws which control them in these movements, how little attention have they paid to the study and investigation of their own bodies, which of all other subjects, I think, they ought to be most interested in to-day. If we examine our literary institutions, we will find that every branch of science is studied, and carefully investigated: the laws which govern the earth, sun, moon, and stars, in their movements, are studied with care; month after month is spent in the study of Greek and Latin, which are only articulate sounds used by common consent, as signs of the ideas of those who lived eighteen hundred years ago, all of which I admit is important and useful. But why is the study of man's own body less important? Why is not a thorough study of the human system introduced into our literary institutions?—Nature's laws cannot be violated with impunity, is it not important, then, that man should be well acquainted with every part of his own system, and its operations, and the laws by which it is governed, that he may observe the laws given him by the All-Wise Creator, and thereby prevent many of the ills to which he subjects himself, by being ignorant of the laws which govern his own system? Again I ask, why not have Anatomy and Physiology thoroughly taught in our literary institutions? Let every one become thoroughly acquainted with the nature and operations of their own systems. Then, and not until then, will they be able to prevent the imposition so much practiced by some who profess great skill in the healing art.

Gentlemen, how often have you witnessed the imposition practiced, not only upon those who are young and inexperienced, and whose means of acquiring knowledge have been limited, but those who have had ample means, and have acquired high literary attainments, but were totally ignorant of the operations of their own systems, and as a consequence, were most egregiously imposed upon by some boaster, with his specifics or cures, performed by supernatural agency which no one possessed but himself. Quackery and humbuggery in the practice of medicine would cease, were the people well acquainted with Physiology and Anatomy. You would no longer see column after column in your daily newspapers filled with advertisements of quack medicines; some of which are too ridiculous to be read or tolerated by a civilized community. Yes, sirs, they are supported and nourished upon the ignorance of the people of their own system, and must and would perish for the want of nourishment, were the people well acquainted with Anatomy and Physiology.

But, gentlemen, the study of Anatomy and Physiology is not only neglected by the people, but is too much neglected by men of our own profession, more especially the latter. It is essential that we be thoroughly acquainted with Anatomy in order to understand Physiology. I am of opinion, that there is no one branch in the study of medicine more important than Physiology, and yet there is no one that is so much neglected.

Why, sirs, there would be as much reason in a man's understanding the management of an engine, and howto repair it when out of order, who knew nothing of the parts of which it is composed, and the action that each part was expected to perform, as for a man to understand the management and regulation of the human system, who was unacquainted with Physiology. And, gentlemen, however excusable the people may be for their ignorance of their own system; or in other words, of Physiology, the man who undertakes the practice of medicine without a thorough knowledge of Anatomy and Physiology, is not only inexcusable, but is, in my opinion, guilty of a high crime.

Gentlemen, suppose a man unacquainted with the parts and operations of an engine, start out on one of our railroads, freighted with his fellow beings, would he not be guilty of a crime? Does he not expose the lives of his fellow men? Are not their lives, to some extent, in his hands? Is he more guilty than he who undertakes the management and regulation of the human system, ignorant of Anatomy and Physiology? Is he any better prepared to repair the human system when in a disordered condition? But, perhaps, you will answer me, of its importance we are all aware, and therefore it needs no argument. But is it true, does the profession to-day, feel the importance of having a thorough knowledge of Physiology? Do they feel its importance to be as essential to their success in the management of disease, as the surgeon feels the necessity of his being thoroughly acquainted with Anatomy? And is not it as closely allied to the management of disease as Anatomy is to operative surgery? The surgeon feels that the life of his patient is in his hands, and that one *mis-stroke* of the knife might sever an artery or some other important part, and send his patient into eternity. Would he excuse himself by saying, that he had done the best he could? that if he had known the artery was there he would not have cut it? No, by no means. He would know that he ought to have known what was there, or else let it alone. He would not nor could make his ignorance an excuse. And I ask, what right has any man to meddle in any way, with the human system, unless he knows what is there—knows every part and the action that each and every part ought to perform? A misapplied remedy, or the neglect to apply an efficient remedy, at the proper time, may be as effectual in sending our patient into eternity, as a mis-stroke of the surgeon's knife, although it may not be so apparent to us or to the world.

How great is the responsibility of the practitioner of medicine? Who can estimate it? How important then, that we not only know every part of the human system, but that we be thoroughly acquainted with the action that every part is expected to perform.

Much of our success in the practice of medicine, or in the management of disease, depends on our being able to distinguish between cause and effect; and in being able to trace the cause from the effect. In many cases the effect is much more apparent than the cause, and if our remedies are applied to the effect, without removing the cause, we will be much like the engineer that would undertake to arrest the onward progress of his engine, by throwing obstructions before its wheels without attempting to remove the power which propelled it onward.

In conclusion, gentlemen, if I have been so fortunate as to have said anything which will stimulate my professional brethren to a more thorough investigation of the human system, or to influence my fellow man to feel a deeper interest in the study of the beautiful mechanism of his own body, my expectations will be more than realized.

